# Eight Letters and Replies—A Communication on Quackery

**Published:** 1878-10

**Authors:** 


### Our Correspondence.

NAVASOTA, TEXAS, Aug. 1878.

DR. UP DE GRAFF:

*Dear Sir*—In the last (July) number, "The Case of Brown," causes me to doubt, where the writer says towards the close that the doctor found Brown had Dyspepsia, and gave him "a little suthin'" which relieved Brown *speedily*, magically; acted like a charm (?). Now I have never seen a case of *Dyspepsia*, which *could* be *relieved* speedily, and I feel very much like asking Ben H. Pratt what it is he gave Mr. Brown. If I knew P's P. O. address, I would write and ask.

I have lately discovered here a common plant, growing in Brazos river swamp, which is possessed of many excellent properties; good for fevers, erysipelas especially, will procure relief from pain, will procure profound sleep *without constipating*, and will act specially as a diuretic. My wife is an invalid, or rather cripple, and suffering with lumbar pains, used morphine. This weed procures sounder sleep than morphine. It acts also as a depurator, or blood purifier, better than sarsaparilla. I obtained a knowledge of its medical properties in curing erysipelas from Indians, through early white settlers, and I have extended its use, and myself found out its other properties.

Our summer is hot, but not as hot as 1877; we have rain nearly every day. Crops are made and laid by. Yours Truly, A. R. K.

The doctor is in error relative to Mr. Pratt's article. The author is not a physician, consequently did not treat the "Case of Brown."

We should like to hear more of the Doctor's newly found medicine, which he recomends so highly. Will he not favor us with his experience in the use of the plant.

---

Here follows a communication from a "medical student" who desires to appear in print. We have read his production twice, and must confess our utter inability to comprehend it. The young man, doubtless, has excellent ideas, but just what they are, cannot be gleaned from his composition. It will be a good gymnastic exercise to grapple with some of his sentences; for this reason we publish it:—

### QUACKERY.

Quackery is the connecting links between morbiness and the gaping graves of our fashionable cemeteries but does not old age death from jeopardous acciends and a score of other difficulties go to make up the tremendious ensnaring chain Yes to the extent of one half but reader men of science talent learning activity have carefully reviewed the fields for the cause of so much of our mortality with the astounding facts that one half can be traced to empirics of medicine those who are dealing out powders pills and portions without the judgement of an innocent baby as to their actions on the human system let alone disease Cities and even small towns and villages are crowded, with these impostors whose banners of stolen M. D. float to the breezes with the enchantment of a shining bait to its intended fellow Advertisments freely strewn of some noted Indian who first learnt his anatomy by scalping the white man and which I am sorry to say his tools not being very excellent he did not make a very mintute investigation He treats diseases of the eye and ear while his instruments for treating and making investigations are simpily a big indian two eyes and a pair of hands He can inform you by general constutional symptoms for the eye whether you have congestion of the retina displacement of same or whether the cause of the trouble with your ear is from inflamation of the drum People as a nation will stop being swindled about the time impostors stop work and which in all probability will be just a few minutes before the last revolution of this earths orb money is the root of all evil yes and a sad one when an impostor of medicine says he shall obtain a livelihood through physics by some means every talent and thought in his power is put at command to entrap the public and prosperity with one (financially) only fires him the brighter to run more poor victimes in his many times gradually embellished den Fools will all be dead about the time people are but half goes with half hence we as as a rational half had better watch off the equilibreum portion and get one fool to obliterate counter poise and thus constitute counter revolution in opposition to past state of offairs sanitaryly again another nomad turns up traveling under the auspices of some foreign F. R. C. S. he he comes to us fresh as the morning air from his past years of confinement in some noted perhaps French Hoteldieu curing the ills of mankind in his patients printed testimonies by crowded scores while a particular hobby is by this man in one branch of his profession that possibly may turn up in the form of tape worms which this great harbinger predestines he can reveal with the audacity Ceasar It has been but a short time since one of these anglicans passed through our vicinity and should he again return a happy audience awaits for his reception and to ameliorate his dealings with physics The world as a people are getting flabby in texture to easily bought to easily sold Swindling is one of the degrading elements nourished in every publics lap but ere where she goes she leaves a sting which by empirics many times proves fatal or a life burden by the satiation in the human system of those poisonousuess remedies that follow them to an earthly doomed grave tired of life to sweet repose in deaths arms Dont tinker if the difficulty is but a slight one go to some reliable source By these means you execute impostors Leave patent nostrums to themselves all remidies it will only be for your benefit in the end many times you are affected with a slight difficulty perhaps loss of appetite a slight tonic and proper diet would soon bring you about then use judgment and go to the correct source and get the remedy reliably prescribed but no some dear old unmeditated unmeaningly friend or aunt thinks Dr. John Hirusetes modified Konkumberances has rought wonders hence the preparation is subjected to trial likewise the system with the no less than thirty nine remidies which it is laboring under while a general harangue scuffle and knock down takes place chemically among the thirty nine as to which is "Chief Cook and Bottle Washer" thus a victim you stand nine chances out of ten to a sick bed and a ever afterwards impaired nutrition Physicians as a whole say little against patent preprations but they are well aware of their being authors of disease and they meet with it practically daily.

---

HONESDALE, PA., Aug. 20, 1878.

DR. UP DE GRAFF:

Can you send me a sure and speedy cure for what is vulgarly called the itch? M.

Yes, we know of a chap here, that will give ten dollars to see you cured. Do not read the sulphur bath squib though before you come.

---

CUBA, N. Y., Sept. 1, 1878.

EDITOR OF BISTOURY:

I am told you can tell me what is good for dyspepsia. If you will, I will be willing to pay whatever you charge. J. M.

Ride a trotting horse every morning, after breakfast, two hours, for six months. Then eat the horse. No charge.

---

POUGHKEEPSIE, N. Y., Aug. 8.

DR. UP DE GRAFF:

*Dear Sir:*—What will take warts off and keep them off? B.

Dig them out with the point of a knife, and throw them into the fire. Otherwise, touch them with *chromic acid*, every day, until they drop out. The last mentioned cure is the best and safest.

---

PITTSBURG, PA., Sept. 4, 1878.

DR. UP DE GRAFF:

Can you tell me what is good for melancholy or the blues. S. M. R.

Yes. Read the letter on quackery in present correspondence. It will either kill or cure you.

Now, right here we must say to our quizzing friends, that if they desire fuller answers to their questions, they must write more fully their symptoms. If our answers seem to be frivolous, at times, it is only because the letters of inquiry are so. We are always willing to give advice when the patient takes pains to describe his case intelligently. Otherwise, you must expect as unsatisfactory replies as are your queries to us.

---

EASTON, PA., Sept. 10, 1878.

DR. UP DE GRAFF:

Can you send us a brace for spinal curvature in a little girl aged ten? We first noticed it to-day and are advised to apply to you. I. D.

We cannot. You must bring the child here for examination, when we can advise you what to do.

---

CANTON, PA., Aug. 25, 1878.

DR. THAD. S. UP DE GRAFF:

*Dear Sir:*—This is to certify that at a meeting of the Canton Literary Society recently held, you were unanimously elected an honorary member of the Society. The thanks of the Society are also hereby tendered you for your very interesting and instructive lecture delivered for their benefit a short time since. Very respectfully yours.

W. T. DAVIDSON, Pres., C. L. Soc.
J. W. PARSONS, Secy.

---

BRUNSWICK, MO., Aug. 11, 1878.

DR. UP DE GRAFF:

*Dear Sir:*—Do you know anything about the use of the plaster of Paris jacket for spinal curvature, and do you recommend its use? I have a child twelve years of age, that is having what the doctors call, a "lateral curvature of the spine." They speak of our using a jacket to correct the trouble, and we seek your advice. B. D.

The jacket referred to is an excellent means of treating certain forms of spinal curvature, but it must be skilfully applied and the patient seen by the surgeon frequently. There is a much better way of remedying the trouble, but the patient must be under daily treatment.

